# Determination of the content of selected elements in medieval waterlogged oak wood from the Lednica Lake—a case study

**DOI:** 10.1007/s11356-017-9972-7

**Published:** 2017-08-26

**Authors:** Magdalena Broda, Marcin Frankowski

**Affiliations:** 1 Institute of Wood Chemical Technology, Faculty of Wood Technology, Poznań University of Life Sciences, Wojska Polskiego 38/42, 60-637 Poznań, Poland; 2 Department of Water and Soil Analysis, Faculty of Chemistry, Adam Mickiewicz University in Poznań, Umultowska 89 b, 61-614 Poznań, Poland

**Keywords:** Waterlogged archaeological wood, Inorganic compounds, IPC-OES, Oak, Submerging environment

## Abstract

The content of selected elements: Al, B, Ba, Ca, Cd, Co, Cr, Cu, Fe, K, Li, Mg, Mn, Mo, Na, Ni, P, Pb, Sb, Si, Ti, V and Zn was determined in archaeological waterlogged oak wood from the Lednica Lake by using the inductively coupled plasma optical emission spectrometry (ICP-OES) method. The concentration of metals (especially heavy metals) in wood is typically specified to characterise this material and evaluate the possibility to use it for industrial purposes. In case of waterlogged archaeological wood intended for further research on new methods of conservation, such an analysis is important for other reasons. As it has been confirmed by numerous conservators and researchers, the presence of metal compounds is a serious problem not only due to their destructive influence on wood tissue but also from a conservation/re-conservation perspective. Metal-containing chemicals may influence conservation treatments by reacting with substances used for wood conservation and causing irreversible damage to wooden objects. Therefore, while developing new solutions for wood conservation, a broad knowledge not only on the state of wood preservation and deterioration but also on interacting chemical factors is required. The results of the research clearly show that content of minerals in waterlogged wood excavated from the bottom of the Lednica Lake considerably exceeded the average percentage of these elements in contemporary wood, which is associated with the mineralisation process. Moreover, variability in metal content was observed between waterlogged and contemporary wood. In waterlogged wood, the highest concentrations of Ca, Fe, Mg and P were observed, while in contemporary oak wood the predominant concentrations of K, Ca, Mn and Si were determined. Statistical analysis showed the variability in content of elements between different archaeological wood zones and contemporary wood. On the basis of the results obtained, it could be concluded that the studied waterlogged wood acted as an adsorbent of elements from water and sediments. High content of metal ions can be an impediment in developing new formulations for conservation, while using this wood as an experimental material. Therefore, while planning to use new chemicals as conservation agents, the possible interactions between chemicals and metals must be taken into consideration.

## Introduction

Wood as a natural lignocellulosic material is effectively decomposed by microorganisms within the carbon cycle in nature. This process is rather fast and usually takes from a few years to several decades. However, wooden objects buried in in the ground or water reservoirs deteriorate very slowly. Due to an extremely low oxygen concentration or even anaerobic conditions in waterlogged terrestrial or aquatic environments, only erosion bacteria or some soft rot fungi are able to decay wood. Slow microbial degradation results in decomposition of cellulose, which is the main component of the secondary cell wall, whereas the lignin-rich middle lamellae remain outwardly intact. The coherent network of unaffected middle lamellae and water, which fills out the degraded cell walls and cell lumina, allow waterlogged decayed wood to keep its physical integrity and the well-preserved appearance as long as it remains wet. Nonetheless, if the wood is allowed to dry, it shrinks, cracks and disintegrates as a result of the impact of the capillary forces upon drying (Björdal et al. 1999; Blanchette 2010; Kim et al. 1996). Once excavated from water reservoirs or from the ground, archaeological waterlogged wooden objects therefore require an immediate treatment to stabilise the degraded wood tissue and protect it from irreversible deformation or destruction.

Typically, chemical and physical characteristics of wood are measured to evaluate its condition and understand mechanisms of degradation. Such a knowledge is essential to design the best possible conservation treatment in order to keep the integrity and dimensions of wooden artefacts. Moreover, it is helpful to suggest the most appropriate environmental conditions for long-term storage or exhibition of waterlogged objects without endangering their safety (Blanchette et al. 1994; Florian 1990). Chemical characterisation of such material is usually limited to qualitative and quantitative analysis of the main organic wood components (cellulose, hemicellulose, lignin) which most commonly deteriorate due to selective enzymatic hydrolysis by anaerobic microorganisms (Bardet et al. 2009; Björdal 2012; Browning 1967; Łucejko et al. 2015; Pizzo et al. 2015; Salanti et al. 2010). However, also abiotic factors, including temperature, pressure and various environmental parameters, can be responsible for wood degradation. Although relatively little is known about the contribution of these processes to wood deterioration due to the long period of time required for evident signs of degradation as well as the masking effect of microbial activity (Blanchette et al. 1994), their influence should not be marginalised. Particularly, the damaging impact of water with its acid-base and oxidising-reducing properties resulting from the presence of metal compounds should not be underestimated.

The presence of some metal ions in wood is considered to have a preservation effect (Blanchette et al. 1994). For example, copper- or boron-based wood preservatives have been widely used for many years to protect timbers exposed outside (Hingston et al. 2001; Obanda et al. 2008). Nonetheless, it has also been repeatedly confirmed that other metal ions can contribute to wood degradation. The products of metal corrosion can extensively degrade wood tissue and cause considerable alterations of a cell wall, including formation of metal pseudomorphs—the replicas of wood cell wall (Baker 1974; Blanchette and Simpson 1992; Marian and Wissing 1960; Parameswaran and Borgin 1980). Problems with metal compounds contributing to archaeological wood degradation are well documented in a number of case studies on shipwrecks such as the Vasa, Mary Rose, Batavia and Oseberg finds—Viking ships (Braovac et al. 2016; Fors and Sandström 2006; Preston et al. 2014). Especially the destructive effect of iron on archaeological wood is well known and confirmed by many researchers (Almkvist et al. 2013; Fors et al. 2012; Huisman et al. 2008; Preston et al. 2014). High iron contents are mainly associated with shipwrecks due to frequent occurrence of iron fastenings or other objects connected with or found near such wooden artefacts. In waterlogged wood, the most common are diverse iron (III) hydroxides and oxides, iron (II) salts as well as reduced sulphur compounds containing iron, such as pyrite and pyrrhotite. Their presence results in mechanical or chemical degradation of wood tissue. Mechanical damage occurs during drying of the wooden object without protective treatment as a result of iron (and other inorganic compounds) accumulating in the surface region. Chemical degradation is rather a post-conservation problem. It proceeds due to oxidation of reduced sulphur species, probably catalysed by iron ions, which generates sulphuric acid that can lead to hydrolysis of wood polysaccharides (Blanchette et al. 1991; Parameswaran 1981; Sandström et al. 2002; Sandström et al. 2005). Coming from the corroded metal objects accompanying wooden artefacts, ionic iron and copper compounds can migrate into the wood and promote wood degradation via radical Fenton reactions (strongly oxidising hydroxy radicals are formed, which can abstract e.g. hydrogen atoms from cellulose or hemicellulose) (Braovac et al. 2016; Henry 2003). Moreover, iron and other metal compounds (Co, Mn, Cu, Ni) can catalyse a variety of different chemical reactions and thus contribute to abiotic wood degradation (Almkvist et al. 2013; Baker 1974; Blanchette and Simpson 1992; Keepax 1975; Sandström et al. 2004).

In waterlogged wood preservation, the presence of metal compounds is a serious problem not only due to their destructive influence on wood tissue but also from a conservation/re-conservation perspective. Metal-containing chemicals in wood may influence conservation treatments. Oxidative reactions involving metal compounds cause depolymerisation of both wood components and preservation agents (e.g. polyethylene glycol). As a result, various low molecular organic acids like glycolic, formic or oxalic are generated, causing further wood degradation (Almkvist et al. 2013). Metal compounds may be located inside the cell wall or precipitated in specific regions of the wood cell, such as bordered pit membranes, where they could influence permeability of the wood and interfere with the conservation process (Florian 1990). They can also interact with tannins in the wood and form insoluble complexes when exposed to oxygen, blocking the wood microstructure for penetration of conservation substances (Sandström et al. 2004). Therefore, the conservator should know precisely the total chemical composition of a wooden object which is to be preserved as well as the location of inorganic substances to consider removing or inactivation of hindering compounds in wood before conservation.

Concentration of metals (especially heavy metals) in wood is typically determined to characterise this material and evaluate the possibility to use it for industrial purposes, e.g. for combustion processes as an ecological energy source or for paper production. It is also useful to observe changes in wood caused by biotic (fungal or insect attack) and abiotic factors (like the influence of environmental contamination on the accumulation of metals in wood). Such a comprehensive analysis of archaeological waterlogged wood has never been done before.

**Fig. 1:**
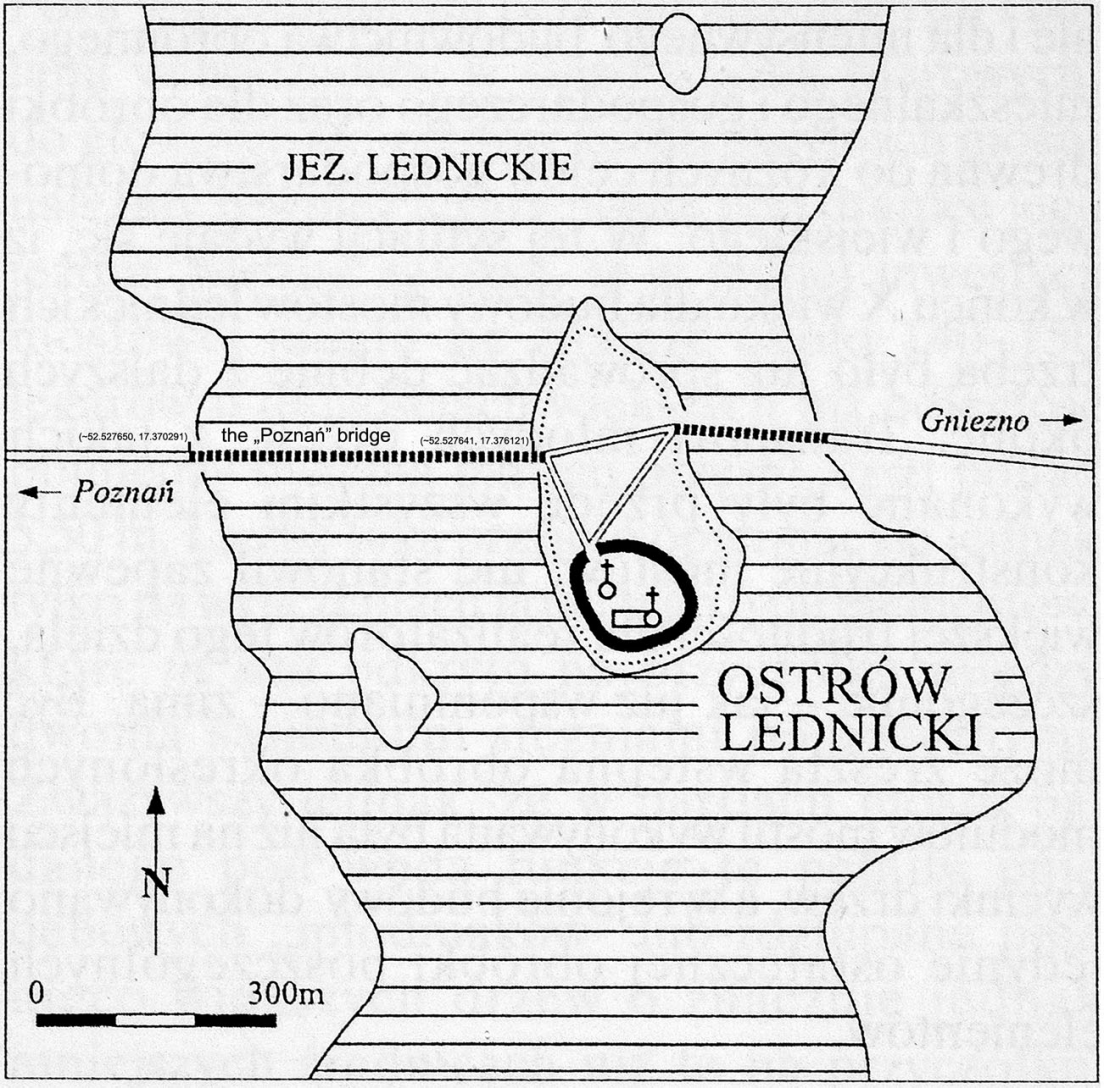
A map of the Ostrów Lednicki Lake with the location of the “Poznań” bridge (Kola and Wilke 2000)

The aim of the research was to evaluate the content of selected metals in archaeological waterlogged oak wood and determine their distribution in respective wood layers. Intended for further study on new methods of wood conservation, this unique wooden material was excavated from the Lednica Lake in the Wielkopolska Region, Poland—one of the most thoroughly explored underwater archaeological sites. The wooden pile was the structural element of the early medieval “Poznań” bridge (Fig. 1). As one of the biggest wooden bridges in Europe at that time, it shows the independent technology of the Slavs from the turn of the tenth and eleventh centuries. The “Poznań” bridge connected the shore of the Ostrów Lednicki island with the road leading into the Poznań city. On the island, there was one of the most prominent political, cultural and religious centres of the monarchy of the first Piasts dynasty during the reign of Mieszko I and his son Bolesław I the Brave, the first King of Poland. Along with wooden remains of the bridge, thousands of early medieval material culture artefacts were found nearby, including the largest unique collection of Slavic militaria (Kola and Wilke 2014). Increased knowledge on the state of archaeological wood preservation, its chemical composition as well as of wood deterioration and interacting chemical, environmental and physical factors is essential for continuing the development of new, improved methods for archaeological waterlogged wood conservation and display.

## Materials and methods

### Samples

The unique archaeological wood material selected for this study was a waterlogged oak (*Quercus robur*) pile. Situated at the bottom of the lake, between other remains of the bridge in a pass with a width of approximately 20 m, the wooden pile was almost completely buried under a layer of mud under anoxic conditions, about 11 m beneath the water surface (Broda et al. 2015). Dating back to the tenth to twelfth centuries, the oak pile contained both sapwood and heartwood. Sapwood (20 to 30-mm thick) was characterised by a light, beige-greyish colour and a soft, disintegrated, spongy structure. Calculated on the basis of conventional wood density (Broda and Mazela 2017), loss of wood substance (LWS) for this part was 78%, which confirms its high degradation level. By contrast, oak heartwood was dark brownish to almost black in colour, with a very firm and hard texture. This part of the wood was preserved very well (Table 1), especially the inner part which remained almost unchanged physically and its structure can be compared to contemporary wood.

**Table 1:** Loss of wood substance of archaeological waterlogged piles

	Waterlogged oak wood			
	S	H1	H2	H3
LWS [%]	78.0	13.8	6.0	1.0

*S* sapwood, *H1* outer heartwood, *H2* intermediate heartwood, *H3* inner heartwood

**Fig. 2:**
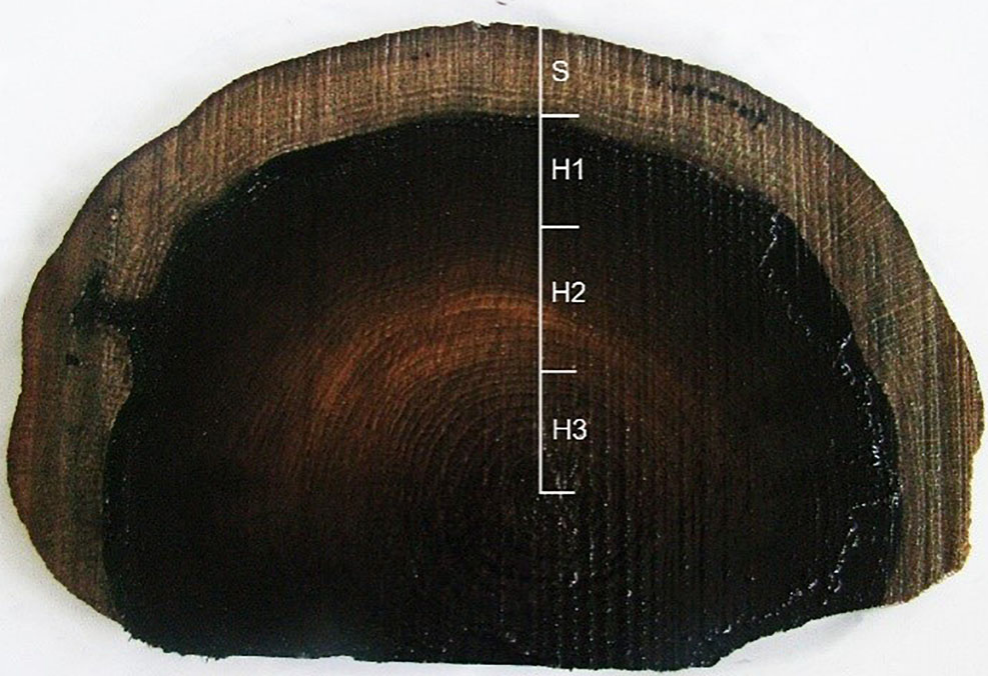
The cross-section of waterlogged archaeological oak wood divided into four zones: S sapwood, H1 outer heartwood, H2 middle heartwood and H3 inner heartwood

The archaeological waterlogged oak pile was cut into 1-cm-thick slices. The slices were subdivided into four zones, differing in the level of wood degradation: sapwood (S) and heartwood: outer (H1), middle (H2) and inner (H3) (Fig. 2). Small square samples (20 × 20 × 10 mm, radial × tangential × longitudinal direction) were cut out from each zone. Additionally, contemporary oak (*Q. robur*) wood from the Wielkopolska Region was examined.

### Methods

#### Sample preparation

Due to the limited availability of archaeological wood, five samples from each zone were used for the analysis. All waterlogged wood samples were freeze-dried using a Lyovac lyophiliser GT2e (Steris, Germany) for 24 h (as well as dry contemporary wood samples). Samples from each zone were milled together on the laboratory cutting mill (MF 10 basic, IKA) to obtain the homogenised powdered mixture. Then 0.500 ± 0.001-g samples were weighed out and poured with 10 ml of 70% nitric acid (Sigma-Aldrich, USA) in 50-ml PTFE containers. The samples were allowed to stay overnight for slow mineralisation. Then the samples were mineralised in a microwave oven (Mars Xpress 5, CEM USA) using a modified EPA 3051 method (Frankowski et al. 2013).

The Sigma-Aldrich (USA) periodic table mix 1 for ICP containing 10 mg L^−1^: Al, As, Ba, Be, Bi, B, Ca, Cd, Cs, Cr, Co, Cu, Ga, In, Fe, Pb, Li, Mg, Mn, Ni, P, K, Rb, Se, Si, Ag, Na, Sr, S, Te, Tl, V and Zn in 10% nitric acid (comprising HF traces) was used for calibration ICP-OES. In order to preserve the standard/sample conditions, the matrix match method was used.

#### Instrumentation

The inductively coupled plasma optical emission spectrometer (ICP-OES) ICPE-9820 (Shimadzu, Japan) with mini-torch was used for qualitative and quantitative detection of selected elements in waterlogged and contemporary oak wood samples. Table 2 presents basic operating conditions of the ICPE 9820. It should be underlined that spectrometer working with mini-torch consumes in total 11.3 L min^−1^ of Ar, with the possibility to reduce the plasma argon to 7.0 L min^−1^ without loss of basic analytical parameters (Zioła-Frankowska and Frankowski 2017). This method allows to reduce cost (low Ar consumption) and time (simultaneous vacuum spectrometer) of analysis and can be applied for routine analysis of samples with different matrix.

**Table 2:** Operating conditions in Shimadzu ICPE-9820 spectrometer for analysis of wood samples

Parameter		Value
Radio frequency power generator		1.2 kW
Gas type		Argon
Plasma gas flow rate		10.0 L min^−1^
Auxiliary gas flow rate		0.6 L min^−1^
Nebulization gas flow rate		0.7 L min^−1^
Plasma view		Vertical torch, axial/radial view
Torch		Mini-torch (quartz)
Nebulizer		Burgener NX-175
Chamber		Cyclone (glass)
Drain		Gravity fed
Injector tube		Quartz (1.2 mm i.d.)
Background correction		2-points
Number of replicates		3
Exposure time		20 s
Plasma viewing conditions		Axial view—all elements Radial view—Ca and Mg
Sample uptake rate		1 ml min^−1^
Spectrometer	Echelle optics	Range of wavelength 167 to 800 nm
	Resolution	≤ 0.005 at 200 nm
	Atmospheric removal system	Rotary vacuum pump ≤ 10 Pa

The SRM 1515 (National Institute of Standards and Technology, USA) certified reference material was used to check the reliability of the ICP-OES measurement. The results obtained for the certified reference material are presented in Table 3 as well as the spectral lines for each element.

**Table 3:** The wavelengths used for element contents determination and the values obtained for SRM 1515 by the ICP-OES analytical technique

Element	Wavelength [nm]	LOD [μg L^−1^]	Values obtained for SRM 1515 by the ICP-OES analytical technique	Certified value (SRM 1515) [μg g^−1^]
Al	167.081	15.9	291 ± 10	286 ± 9
B	249.773	0.69	26 ± 2	27 ± 2
Ba	455.403	0.17	48 ± 1	49 ± 2
Ca (%)	396.847	0.78	1.518 ± 0.011	1.526 ± 0.015
Cd	226.502	0.30	–	–
Co	228.616	0.49	–	–
Cr	205.552	0.42	–	–
Cu	324.754	0.96	5.50 ± 0.22	5.64 ± 0.24
Fe	259.940	0.11	–	–
K (%)	766.490	2.29	1.65 ± 0.04	1.61 ± 0.02
Li	670.784	0.09	–	–
Mg (%)	279.553	0.95	0.270 ± 0.006	0.271 ± 0.008
Mn	257.610	0.04	55 ± 1	54 ± 3
Mo	202.030	0.87	0.090 ± 0.011	0.094 ± 0.013
Na	588.995	3.10	25.0 ± 1.1	24.4 ± 1.2
Ni	231.604	0.58	0.89 ± 0.10	0.91 ± 0.12
P	177.499	21.1	–	–
Pb	216.999	3.18	0.462 ± 0.032	0.470 ± 0.024
Sb	206.833	4.48	–	–
Si	251.611	0.24	–	–
Ti	334.941	0.33	–	–
V	292.402	0.29	0.27 ± 0.02	0.26 ± 0.03
Zn	213.856	0.33	12.7 ± 0.2	12.5 ± 0.3

## Results and discussion

The content of elements mentioned in the previous section was determined in archaeological and contemporary oak wood samples. The results expressed as microgram per gram are presented in tables below. Co, Cr, Li, Mo, Ti and V content were also determined but were below the limit of detection of the analytical technique.

In waterlogged wood, the highest concentrations were observed for Ca, Fe, Mg and P (Table 4). Particularly high content was observed for Ca ions ranging between 4756 μg g^−1^ for oak inner heartwood and 6255 μg g^−1^ for oak sapwood, which was about 12–16 times higher than in contemporary oak wood. Such high accumulation of this element in waterlogged wood tissue results from the specific conditions of the bottom of the Lednica Lake, the excavation site of the wooden pile. The lakebed is covered by a layer of slurry mud which is an early stage of a newly formed calcareous gyttja, overlying a sandy detritus-calcareous gyttja, containing a large number of mollusc shells (Kola and Wilke 2000). Calcium carbonate content in sediments of this type ranged between 25 and 50% (Tobolski 1991).

**Table 4:** Ca, Fe, Mg and P content in oak wood

Sample	Element content [μg g^−1^]			
	Ca	Fe	Mg	P
S	6255 ± 51	3820 ± 35	280.5 ± 3.3	420.5 ± 7.5
H1	5672 ± 43	4351 ± 45	268.5 ± 3.7	288.4 ± 5.2
H2	6223 ± 55	3180 ± 30	318.5 ± 4.2	333.4 ± 6.6
H3	4756 ± 32	1583 ± 12	194.2 ± 2.0	18.79 ± 0.42
CO	393.3 ± 3.6	2.504 ± 0.178	< LOD	13.69 ± 0.35

*S* archaeological oak sapwood, *H1–H3* archaeological oak heartwood (outer, middle and inner, respectively), *CO* contemporary oak

Quite a high concentration of iron was also observed in waterlogged oak wood samples, varying from 1583 μg g^−1^ for H3 to 4351 μg g^−1^ for H1. It can be explained by the presence of a greater number of iron compounds in the burial environment (water and sediments) resulting from an enormous number of corroded medieval weapons found in the lake, especially within the area of the remains of the “Poznań” bridge (Kola and Wilke 2014). Moreover, the oak wood is especially susceptible to iron accumulation due to the high concentration of tannins. Tannins react with iron forming stable chemical compounds, which turns natural wood colour into dark brownish to almost black (it can be observed in the cross-section of an oak pile—see Fig. 2). Similar concentrations of iron were determined by the X-ray photoelectron spectroscopy in wooden elements of the Mary Rose warship, ranging from below 1000 μg g^−1^ in light-coloured wood to over 7000 μg g^−1^ in “black oak” (Sandström et al. 2005). Coming from corroding bolts, nails and other objects of mild steel present in oak construction of the ship, together with sulphur compounds they caused chemical wood degradation and probably also contributed to incremental degradation of PEG polymers used then for ship conservation (Sandström et al. 2005). Contemporary oak wood contained only 2.504 μg g^−1^ of iron ions.

Concentrations of two further nutrient elements, Mg and P, were at a similar level in the outer and middle parts of waterlogged oak wood, ranging between 270 and 420 μg g^−1^. The inner, almost non-degraded part of wood contained a smaller amount of those elements. In case of Mg content, it was about 194.2 μg g^−1^, while in contemporary wood it was below the limit of detection. P content in the stem part was 18.79 μg g^−1^ and was comparable to its amount determined in contemporary wood (13.69 μg g^−1^). Magnesium compounds in lake water and sediments mainly come from plant putrefaction. Mineral compounds of surrounded soils can also be a source of this element. By contrast, the phosphorus content has its roots primarily in intensification of agricultural production and, more precisely, in the intensive phosphorus fertilisation of encompassing cultivated lands. This element plays a crucial role in the eutrophication of inland waters (Reddy et al. 1999).

Five heavy metals content: cadmium (Cd), copper (Cu), manganese (Mn), lead (Pb) and zinc (Zn) in oak wood was also determined. The highest concentration was recorded for Mn (34.01–59.28 μg g^−1^) and Pb (4.022–9.713 μg g^−1^). Zn and Cu were at a quite similar level about 1.116–7512 μg g^−1^ with the exception of sapwood part where higher concentrations of elements were observed: 5.038 μg g^−1^ of Cu and 14.97 μg g^−1^ of Zn. Lower concentration was found for Cd—about 0.2–0.6 μg g^−1^. Heavy metals are introduced into the environment as a result of natural processes occurring in nature but also due to human activity, mainly industrial development. The presence of agricultural surroundings of the Lednica Lake protected it from pollution with heavy metals, which is visible in their low concentration in water (Table 5). The increased content of heavy metals in sediments (Table 5) is characteristic for lakes in northern Poland. It can be explained mainly by the presence of numerous mollusc remains at the bottom of the lake, forming calcareous gyttja. This kind of organism can absorb metals from water and accumulate them in their tissues, enriching the lake sediments with these elements (Baršytė Lovejoy 1999). From there, over the centuries, thanks to microbial activity, they diffused into water and then into wood tissue of the construction elements of the bridge, present in the mud at the bottom of the lake (Wood 1987).

**Table 5:** Cd, Cu, Mn, Pb and Zn concentration in oak wood and water and sediments of the Lednica Lake

Sample	Element content [μg g^−1^]				
	Cd	Cu	Mn	Pb	Zn
S	0.565 ± 0.006	5.038 ± 0.069	34.01 ± 0.29	8.399 ± 0.122	14.97 ± 0.02
H1	0.598 ± 0.008	1.393 ± 0.014	59.28 ± 0.84	9.713 ± 0.143	2.751 ± 0.003
H2	0.371 ± 0.004	1.162 ± 0.011	57.71 ± 0.72	8.138 ± 0.111	2.292 ± 0.003
H3	0.171 ± 0.002	1.223 ± 0.011	39.21 ± 0.51	4.022 ± 0.092	1.116 ± 0.002
CO	<LOD	1.501 ± 0.014	71.43 ± 0.92	0.218 ± 0.007	0.429 ± 0.003
Water [μg ml^−1^ l]^a^	n.a.	0.027 ± 0.002	0.056 ± 0.006	<LOD	0.056 ± 0.001
Sediment [μg g^−1^]	1.58^b^	38.89^a^	103.42^a^	71.5^b^	147.59^a^

*S* archaeological oak sapwood, *H1–H3* archaeological oak heartwood (outer, middle and inner, respectively), *CO* contemporary oak, *n.a.* not analysed

^a^Rybak et al. 2013

^b^Cieslewicz and Rózanski 2010 (average content of metals determined on the basis of the measurements of sediments from 13 lakes of northern Poland)

Concentrations of other measured elements in waterlogged and contemporary oak wood are shown in Table 6. The highest concentration of potassium was observed in contemporary wood. This is a normal trait. Potassium plays a very important role in many metabolic processes and it is one of the most common elements in fresh wood (Kollman and Côté 1968). A relatively high potassium content in waterlogged wood can be explained by high accumulation of this element in lake sediments. The Lednica Lake is surrounded by farmland. Potassium, a very common component of fertilisers, is considered an indicator of the intensity of agricultural use of the catchment. Potassium content in sediments of similar lakes in the Masurian Lake District was 1.01 to 1.84 g kg^−1^ (Rafałowska and Sobczyńska-Wójcik 2014). Comparable results were obtained for sediments of the Masurian lakes surrounded by farmland and forest, where potassium content was between 0.66 and 1.31 g kg^−1^ (Szyperek 2005). In case of waterlogged wood, the highest content was observed for Si, ranging from 59.45 μg g^−1^ in sapwood to 147.1 μg g^−1^ in heartwood. Only small concentrations of Al, B, Ba Na, Ni and Sb were observed in both waterlogged and contemporary oak wood.

**Table 6:** Concentration of Al, B, Ba, K, Na, Ni, Sb and Si in oak wood and water and sediments of the Lednica Lake

Sample	Element content [μg g^−1^]							
	Al	B	Ba	K	Na	Ni	Sb	Si
S	11.43 ± 0.13	19.38 ± 0.33	15.69 ± 0.11	64.25 ± 0.11	24.79 ± 0.32	0.921 ± 0.011	3.138 ± 0.044	59.45 ± 0.78
H1	6.182 ± 0.091	23.51 ± 0.38	18.42 ± 0.15	40.05 ± 0.08	12.01 ± 0.23	0.911 ± 0.015	1.829 ± 0.022	147.1 ± 2.28
H2	3.633 ± 0.053	15.37 ± 0.24	7.895 ± 0.07	89.79 ± 0.15	12.24 ± 0.21	0.731 ± 0.009	2.681 ± 0.37	125.1 ± 1.79
H3	2.015 ± 0.032	11.01 ± 0.19	14.96 ± 0.11	33.36 ± 0.08	33.16 ± 0.36	0.398 ± 0.005	0.145 ± 0.002	130.7 ± 1.87
CO	1.163 ± 0.022	3.416 ± 0.044	13.32 ± 0.12	500.4 ± 6.2	3.464 ± 0.49	0.083 ± 0.001	0.703 ± 0.009	70.27 ± 0.92

*S* archaeological oak sapwood, *H1–H3* archaeological oak heartwood (outer, middle and inner, respectively), *CO* contemporary oak

**Fig. 3:**
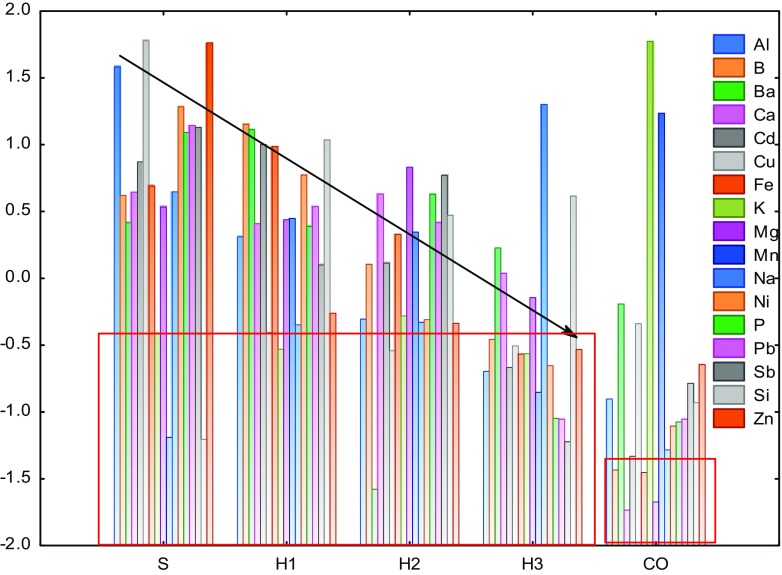
The standardised results for the data set of the ICP-OES analysis for different zones of waterlogged wood (S sapwood, H1–H3 heartwood) and contemporary oak (CO) wood

The results of the ICP-OES analysis were standardised to visualise variability in the content of elements between different archaeological wood zones and contemporary wood, and the results for the whole data set are presented in Fig. 3. The Statistica 12.5 data analysis software system was used for data standardisation, the factor analysis and the principal component analysis (PCA) analyses (Stat Soft, Inc.). For archaeological waterlogged wood, a correlation between changes of elemental concentration and the degree of wood degradation (or loss of wood substance—see Table 1) in the respective wood zones (S, H1–H3) was observed, which is marked with an arrow in Fig. 3. In most cases, the concentration of metals increases as the loss of wood substances rises from the core outward. Chemical compounds usually undergo deposition to bottom sediments. Wooden piles, dipped in sediments, slowly absorb the elements from the environment and accumulate them in wood tissue. The higher the degree of wood degradation, the less dense and more porous is the wood tissue, and the easier the penetration of metal ions. Oak wood is characterised by high density of about 710–900 kg m^−3^ in the dry state and is generally hard to impregnate. The most degraded, sponge-like sapwood zone is the most accessible for accumulation of ions, while the almost non-degraded inner heartwood part retains properties of fresh wood and remains low permeable, which was visible in the results obtained.

In case of contemporary wood, a variation in metal content was observed in comparison with waterlogged wood. In general, content of minerals in waterlogged wood considerably exceeds the average percentage of these elements in contemporary wood, which is associated with the mineralisation process frequently observed for archaeological wood excavated both from the ground and water reservoirs (Hoffmann 1982; Grattan and Mathias 1986). The exceptions are K and Mn contents which were significantly higher in contemporary wood. This could be explained by their leaching from waterlogged wood and then the usage of those minerals as nutrients by water plants and algae. Potassium is a very important element for plant cells. It is an activator of more than 50 enzymes and thus actively participates in the processes of respiration and photosynthesis. It also regulates water economy and supports cell growth. Together with calcium and magnesium, it is one of the most common elements in ashes from wood (Kollman and Côté 1968). Manganese also plays a very important metabolic function in plants. Its concentration in fresh wood can vary over a broad range, depending on the species, age and part of tree as well as on the manganese concentration in soil (Kabata-Pendias and Pendias 1999).

**Fig. 4:**
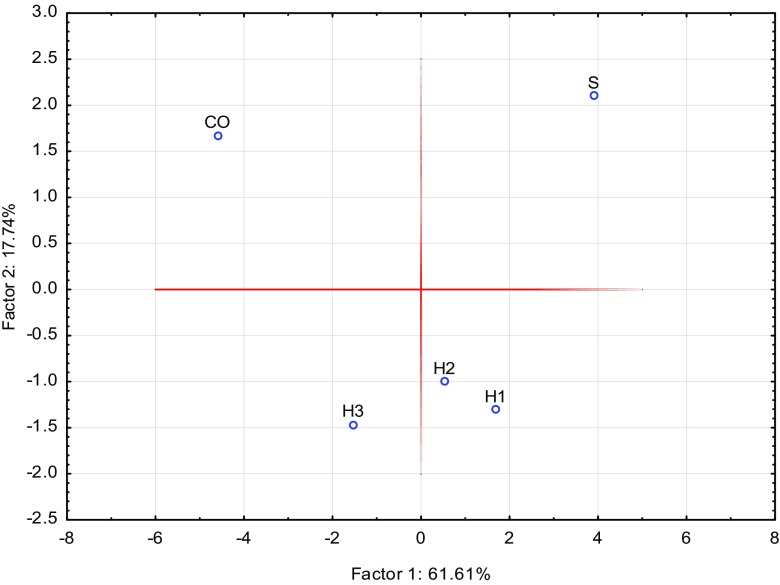
The principal component analysis for the dataset of the ICP-OES analysis for different zones of waterlogged wood (S sapwood, H1–H3 heartwood) and contemporary oak (CO) wood

The principal component analysis was performed for the whole data set obtained to display similarities between wood zones and to compare archaeological waterlogged wood with contemporary wood. The results of the analysis (Fig. 4) clearly illustrate the process of metal accumulation in waterlogged wood, especially in the heartwood zones H1–H3. However, based on the PCA, the deposition of metals in H1–H3 parts through the sapwood zone was confirmed. It should be underlined that adsorption of metals within the S zone and absorption into H1–H3 shows changes indicating the possible “mechanism” of accumulation in waterlogged wood. Besides, a different relation for the CO sample was found, which is strongly connected with different external conditions that affect fresh wood (e.g. different content of metals in the environment).

In order to show the mechanism of absorption of the elements by wood, a comparison of the correlation between the elements evaluated for archaeological oak wood was made. To this end, the factor analysis was performed (Table 7). On the basis of the three factors, the analysis found only a negative correlation between Ba, Mn and Si and a group of other metal ions. Elements such as B, Cd, Fe, Ni and Pb are in the first group with the highest correlation (loads > 0.7). These are elements derived from natural processes occurring in the environment, deposited in the lake mainly due to their run-off from the drainage basins. The second group consists of Al, Cu and Zn coming from the natural weathering processes, the run-off from the surrounding fields and as a result of rainfall. In contrast, the third group contains the elements related mainly to agricultural activities on the catchment area, as Ca, K and Mg are the common components of fertilisers.

**Table 7:** Matrix of factor loadings calculated on the basis of elements concentration in archaeological oak wood

Element	Factor loadings (varimax, normalised) (marked loads are > 0.700)		
	Factor 1	Factor 2	Factor 3
Al	0.649	0.750	0.124
B	0.999	− 0.020	0.013
Ba	0.524	0.184	− 0.831
Ca	0.519	0.271	0.810
Cd	0.968	0.191	0.161
Cu	0.315	0.944	0.095
Fe	0.963	0.029	0.265
K	− 0.023	0.076	0.996
Mg	0.460	− 0.047	0.886
Mn	0.353	− 0.881	0.313
Na	− 0.629	0.542	− 0.556
Ni	0.844	0.534	0.002
P	0.675	0.346	0.651
Pb	0.768	0.372	0.519
Sb	0.547	0.374	0.748
Si	− 0.072	− 0.962	− 0.261
Zn	0.365	0.915	0.166

## Conclusions

The results of the presented research clearly show that archaeological oak wood excavated from the bottom of the Lednica Lake contained high quantities of metals. In general, waterlogged wood contained considerably higher concentrations of these elements than contemporary oak wood, which is associated with the mineralisation process of archaeological wood. Furthermore, variability in metal content was observed between waterlogged and contemporary wood. The highest concentrations of Ca, Fe, Mg and P elements (about 5700 and 3200 μg g^−1^ for Ca and Fe, respectively, and about 260 μg g^−1^ for Mg and P) were determined in archaeological wood samples while in contemporary oak wood the predominant concentrations of K (about 500 μg g^−1^), Ca (about 390 μg g^−1^), Mn and Si (about 70 μg g^−1^) were observed. The principal component analysis revealed the mechanism of accumulation of metals in waterlogged wood—the accumulation through the most degraded and porous sapwood zone to inner heartwood layers. In turn, the factor analysis allowed for identification of three groups of elements deposited in researched wood: metals derived from natural processes occurring in the environment, run-off to the lake from the drainage basins (like B, Cd, Fe, Ni and Pb), the elements from the natural weathering processes, leached out from the surrounding fields by rainfall (as Al, Cu and Zn) and metals related mainly to agricultural activities, as Ca, K and Mg. This analysis showed also only a negative correlation between Ba, Mn and Si and a group of other metal ions.

On the basis of the results of this case study, it could be concluded that waterlogged wood acts as an adsorbent of elements from water and sediments. Therefore, high content of metal ions in such kind of wood may be expected. This can be an impediment in developing new formulations for conservation, while using this kind of wooden artefacts as an experimental material. Therefore, while planning to use new chemicals as conservation agents, the possible interactions between chemicals and metals must be taken into consideration.
